# Wear Behavior of Aluminium Metal Matrix Composite Prepared from Industrial Waste

**DOI:** 10.1155/2016/6538345

**Published:** 2016-02-18

**Authors:** L. Francis Xavier, Paramasivam Suresh

**Affiliations:** Department of Mechanical Engineering, Karpagam College of Engineering, Myleripalayam, Othakalmandapam, Coimbatore, Tamil Nadu 641 032, India

## Abstract

With an increase in the population and industrialization, a lot of valuable natural resources are depleted to prepare and manufacture products. However industrialization on the other hand has waste disposal issues, causing dust and environmental pollution. In this work, Aluminium Metal Matrix Composite is prepared by reinforcing 10 wt% and 20 wt% of wet grinder stone dust particles an industrial waste obtained during processing of quarry rocks which are available in nature. In the composite materials design wear is a very important criterion requiring consideration which ensures the materials reliability in applications where they come in contact with the environment and other surfaces. Dry sliding wear test was carried out using pin-on-disc apparatus on the prepared composites. The results reveal that increasing the reinforcement content from 10 wt% to 20 wt% increases the resistance to wear rate.

## 1. Introduction

Wet grinder is one of the vital household appliances used by south Indians to prepare batter by grinding the raw materials like rice and dhal. Generally, these wet grinder stone dust particles are used in manufacturing various construction admixtures, used as land-fillers, dumped in agricultural lands, or discarded into the environment, leading to serious problems relating to the dust pollution and environmental deterioration. The main health effects associated with dust exposure are asthma and bronchitis and in the utmost cases it causes cancer. Therefore, efforts are to be taken to control environmental pollution arising due to disposal of these industrial wastes by converting them into utilizable raw materials for real time usable applications.

In this investigation, Aluminium Metal Matrix Composite (AMMC) is prepared by reinforcing the wet grinder stone dust (WSD) particles, an industrial waste generated during the stone dressing operation of the quarry rocks. AMMCs are extensively used in many industrial applications, defense, aerospace, and sports equipment because of many admirable properties like high strength, high stiffness, high thermal conductivity, and combined properties like wear resistance with fracture toughness and high strength with corrosion resistance [1]. On the other hand, the application of AMMC is restricted due to poor wear resistance under dry lubrication conditions [2–4].

Wear is one of the most frequently encountered industrial issue where the material is affected mainly by speed, environmental conditions, and working load [5]. Wear is a slow and progressive loss of material which are subjected to repeated rubbing action. Wear causes an enormous amount of expenditure by repairing or replacing the worn-out parts or equipment [6]. The wear resistance of metal matrix composite depends mainly on various microstructural characteristics like particle size, volume fraction, distribution of reinforcement material, and shape [7–11]. Among the different types of reinforcement, particulate form of ceramics reinforced with AMMC has desirable and attractive properties like ease of fabrication and can withstand higher operating temperature and oxidation resistance compared to other geometries of reinforcement such as fiber and flakes [12].

As there is an increasing demand for low cost reinforcement in preparing the composites [13], in this research work an attempt has been made to prepare AMMC suitable for use in engineering applications where wear properties are desired.

## 2. Materials and Methods 

In this present investigation, Al6063 alloy is used as the matrix material to prepare the composite. Al6063 alloy is typically used in aircraft, extrusions, architectural applications, window frames, and irrigation tubing [14]. The chemical composition of Al6063 alloy (%) is Si = 0.303, Fe = 0.071, Cu = 0.0050, Mn = 0.0759, Mg = 0.529, Cr = 0.0037, Ni = 0.014, Zn = 0.025, Ti = 0.0151, Ca = 0.0146, and Al = 98.94.

The composites were prepared by two-step stir casting process. Initially the WSD particles were preheated up to 250°C in order to improve its wettability with the base matrix alloy and eliminate the moisture content. Then the Al6063 alloy was charged into the crucible which is fitted with a temperature probe and heated to a temperature of 780°C in order to melt the alloy. Then the liquid alloy was allowed to cool until it attains semisolid state. The preheated WSD particles along with 0.1 wt% magnesium were added to the melt which is in the semisolid state and stirred manually for 15–20 minutes. The temperature was then raised to 850°C and a second stirring was done for 15 minutes. Finally the prepared composite was poured into the prepared sand molds.

In this work, three samples were prepared with the following combination: sample-I = Al6063 alloy, sample-II = Al6063 alloy + 10 wt% of WSD particles of 45 micron of particle mean size, and sample-III = Al6063 alloy + 20 wt% WSD particles of 45 micron of particle mean size.

**Figure 1:**
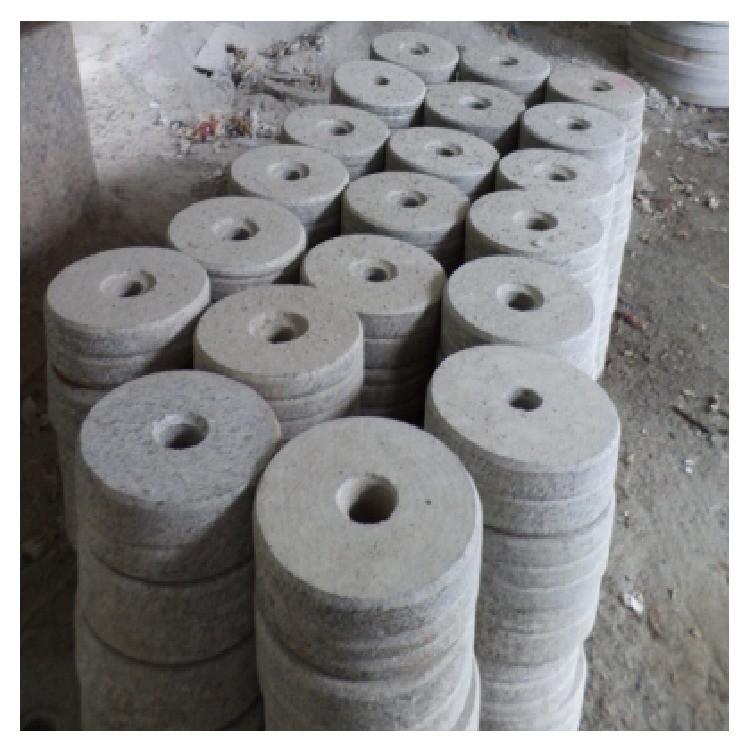
WSD processing unit.

**Figure 2:**
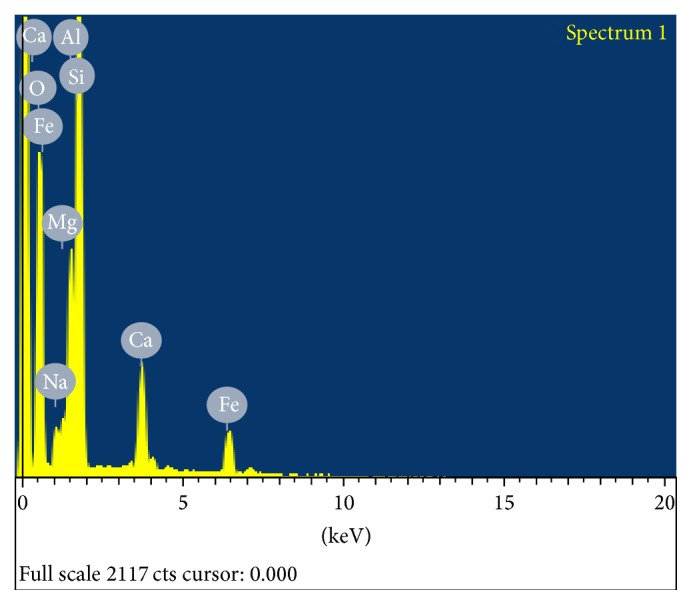
EDAX test report of WSD dust particles.

**Figure 3:**
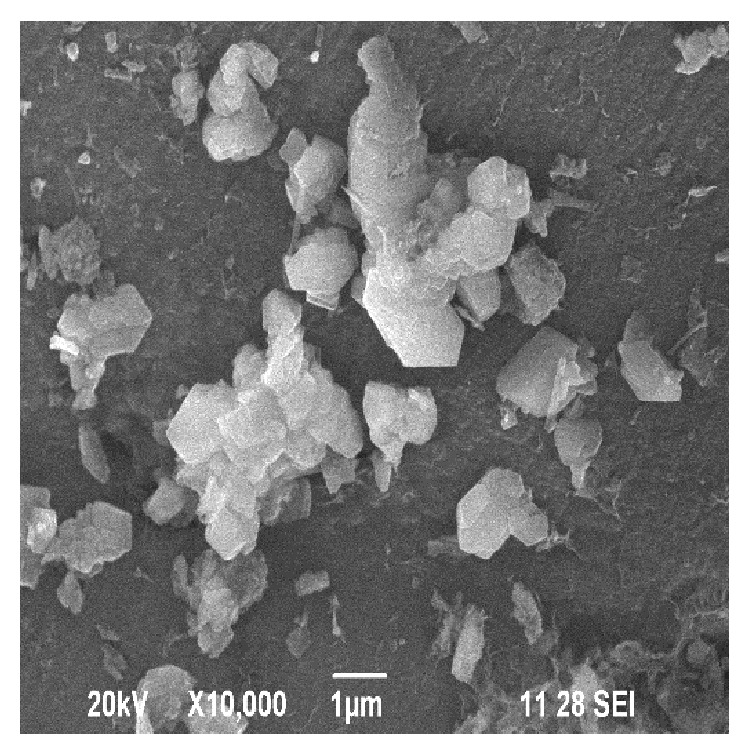
SEM of WSD dust particles.

**Figure 4:**
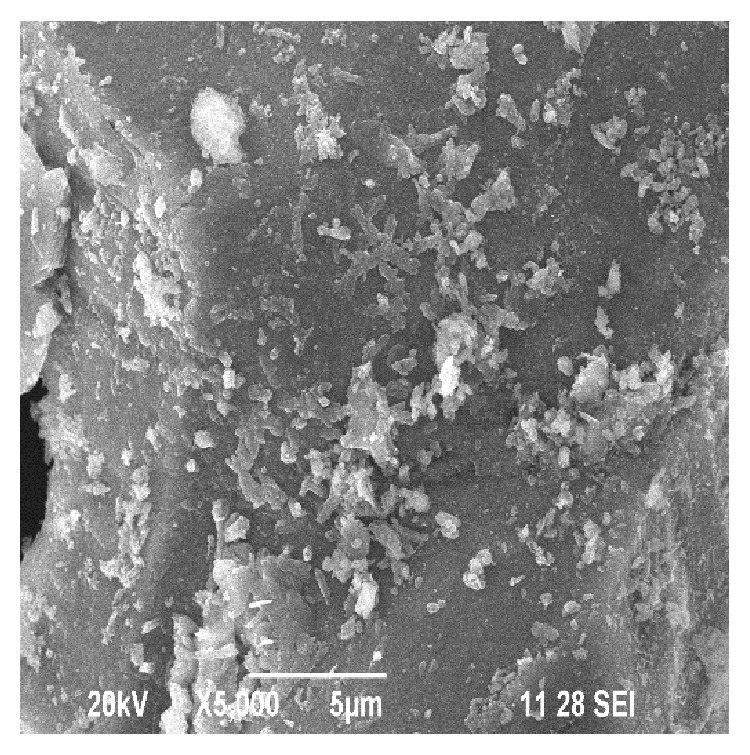
SEM of specific surface of WSD dust particles.

The WSD processing unit is shown in Figure 1. The surface topography of the WSD particles was studied using SEM and EDAX analysis.  Figure 2 shows the EDAX test report of WSD particles revealing the presence of Si, Ca, Fe, Al, Mg, and Na as its major elements. Figures 3 and 4 show the SEM image and specific surface of the WSD particles.

**Figure 5:**
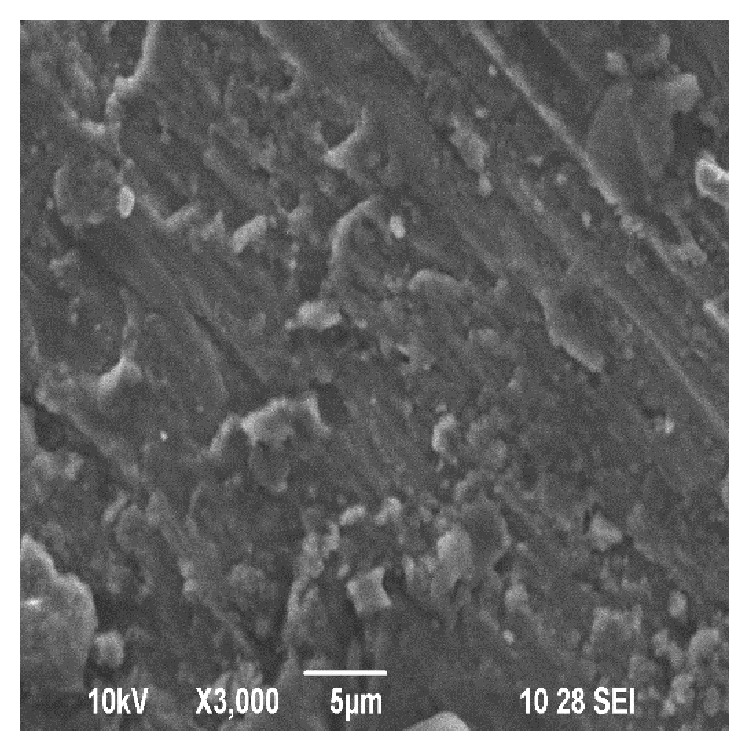
SEM of sample-II (10 wt% of WSD particles).

**Figure 6:**
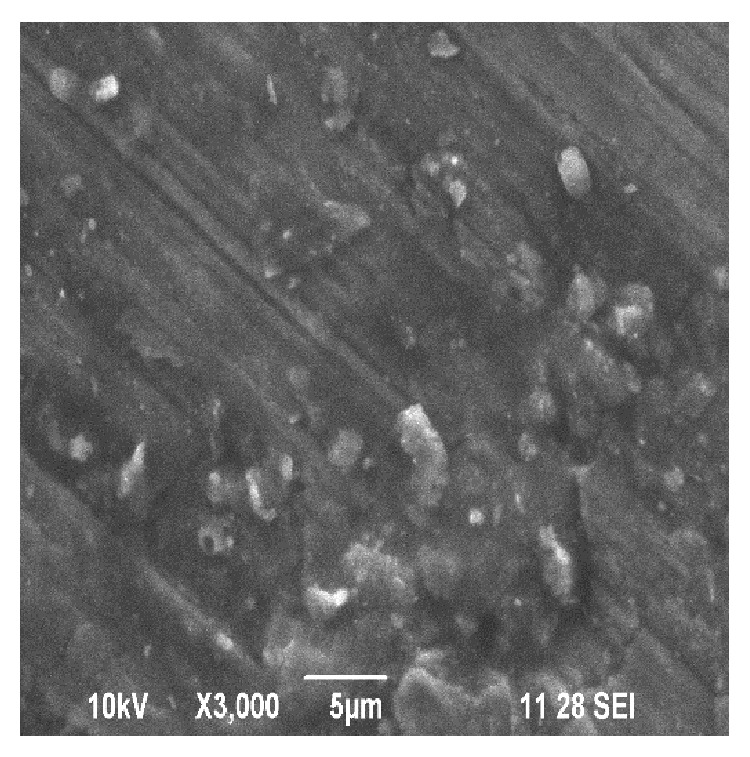
SEM of sample-III (20 wt% of WSD particles).

Figures 5 and 6 show the SEM image of the distribution of WSD particles in the prepared sample-II and sample-III, respectively. The distribution of the WSD particles can be seen approximately uniformly in the matrix.

### 2.1. Mechanical Properties of the Prepared Samples

The hardness and tensile test was conducted on the prepared composite materials in order to study its mechanical properties.  Table 1 shows the results of the hardness, yield strength, ultimate tensile strength, and elongation (%) of the samples taken for the investigation. According to the hardness test results, the average hardness of the specimens prepared by reinforcing 10 wt% and 20 wt% of industrial waste was measured as 62 BHN and 74 BHN, respectively. The hardness values of the prepared composites were found to be greater than that of the Al6063 alloy. The hardness of the prepared composite increases with increase in the reinforcement content. This may be attributed to the presence of hard reinforcements like Al_2_O_3_ particles in the WSD particles.

It can be observed from Table 1 that the yield strength and tensile strength of sample-II and sample-III are higher than that of sample-I. The highest tensile strength values were obtained for the composite prepared by reinforcing 20 wt% of WSD particles. Increasing the reinforcement content from 10 wt% to 20 wt% increases the yield strength, hardness, and tensile strength of the composites but reduces its ductility. This may be due to poor bonding and agglomeration of the reinforced particles with the Aluminium matrix.

### 2.2. Experimental Setup for Wear Test

Pin-on-disc test apparatus (Model TR 20-LE, Ducom) was used to investigate the dry sliding wear characteristics of the composites as per the ASTM G99 Standards. The specimens were machined to pin size of 10 × 10 × 25 mm. The wear loss was measured directly as the height loss of the specimen using Linear Variable Differential Transducer (LVDT). During the test the specimen was pressed against the rotating EN32 steel disc with hardness 65 HRC by applying the load. The frictional force was also recorded during each test. The coefficient of friction was determined by dividing friction force with the normal load [15]. The test was conducted at normal room temperature with the load ranging from 9.81, 19.62, and 29.43 N at a sliding speed of 1.57, 3.14, and 4.71 m/s and with a sliding distance of 1000, 2000, and 3000 m. The levels and the values of the parameters (load, sliding speed, and sliding distance) used for conducting the wear test were determined by conducting preliminary experiments.

## 3. Results and Discussions

### 3.1. Coefficient of Friction and Wear Rate

**Figure 7:**
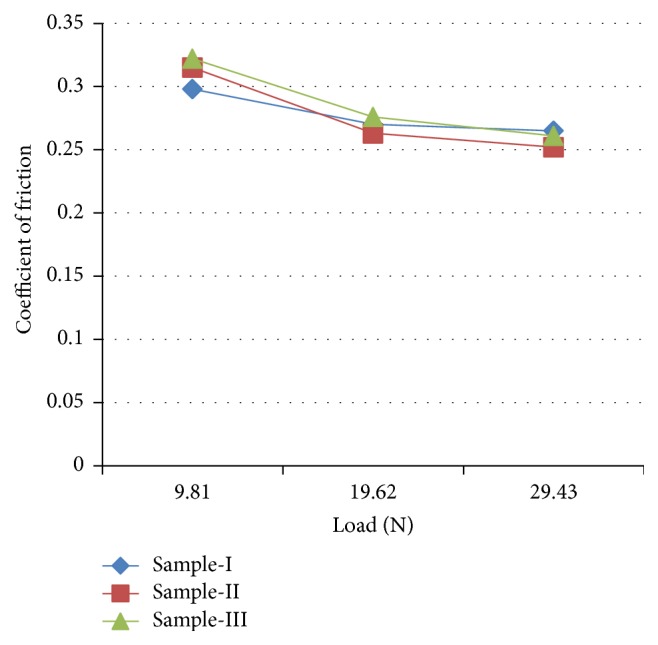
Variation of coefficient of friction with applied load.

**Figure 8:**
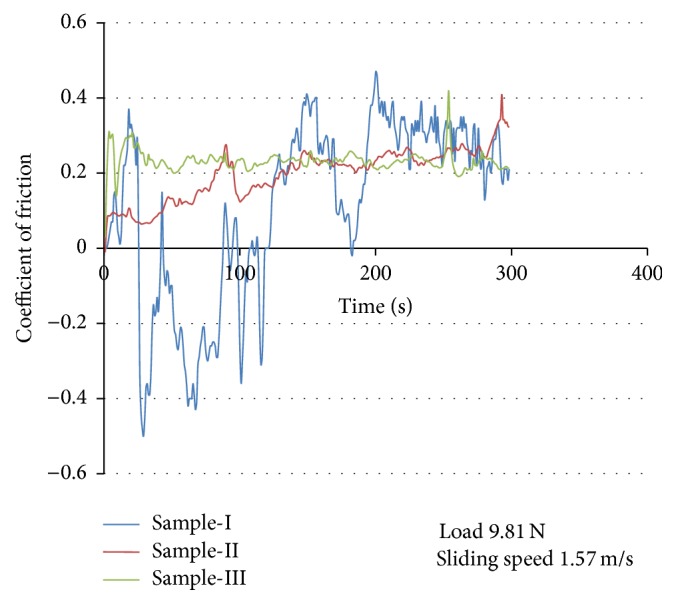
Variation of coefficient of friction with time.

Friction between the abrasive steel disc and the composite pin plays a vital role in the dry sliding wear behavior [16]. In general, friction is the result of three components: ploughing, adhesion, and asperity deformation [17]. In this investigation, the frictional force (*F*) in N was measured directly from the wear apparatus. By using the friction force (*F*) value, the coefficient of friction was calculated by dividing the frictional force (*F*) by the normal load [15]. The coefficient of friction is an important parameter which is used to evaluate the wear resistance of the materials [18].  Figure 7 shows the variation of coefficient of friction (COF) with applied load. It is clear from Figure 7 that the COF reduces with increase in the applied load. This may be due to softening of the composite surface caused due to frictional induced heat between the sliding surfaces [19]. Thus the COF drops down steadily with increase in the applied load.  Figure 8 shows the variation of COF with time for the samples taken for the investigation.

The COF follows a sinusoidal wave pattern. The rise and fall of the COF values may be due to the cyclic formation and removal of the material from the steel disc onto the pin surface. In case of sample-I, the COF initially increases steadily after which there is a drop in the COF and then increases. From the variation of the COF, it is clear that the initial wear mechanism for sample-I is adhesive, which converts to abrasive at a later stage.

The presence of wear debris and ploughing marks in the SEM report of the worn surface of the samples as shown in Figures 10(a), 11(a), and 12(a) supports the adhesive and abrasive wear mechanism. It is clear from Figure 8 that the COF for both sample-II and sample-III increases rapidly during the initial stage and reaches a steady state after 200 seconds. The initial rapid raise in COF for the composites may be due to the direct contact between the hard reinforcement and the rotating disc.

**Figure 9:**
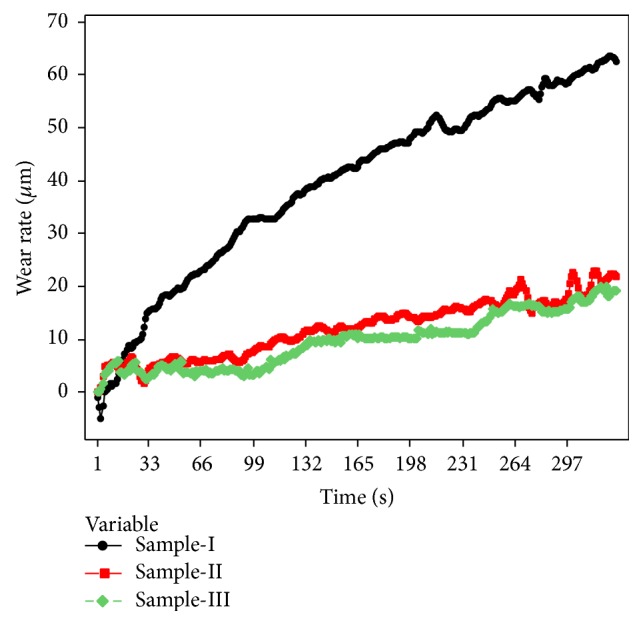
Variation of wear rate of the samples with time after running 1000 m at 1.57 m/s sliding speed and at an applied load of 9.81 N.

**Figure 10:**
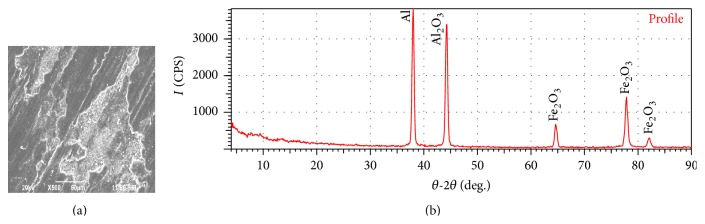
(a) SEM of worn surface of sample-I after running 1000 m at 29.43 N load and 4.71 m/s sliding speed. (b) XRD test report of the worn surface of sample-I.

**Figure 11:**
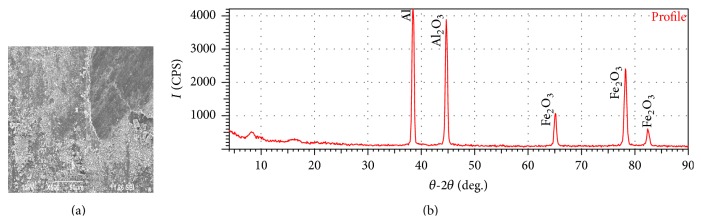
(a) SEM of worn surface of sample-II after running 2000 m at 29.43 N load and 4.71 m/s sliding speed. (b) XRD test report of the worn surface of sample-II.

**Figure 12:**
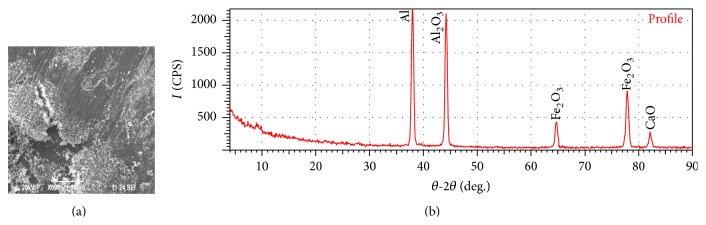
(a) SEM of worn surface of sample-III after running 3000 m at 29.43 N load and 4.71 m/s sliding speed. (b) XRD test report of the worn surface of sample-III.

Figure 9 shows the results of the variation of wear rate of the samples with time after running 1000 m, at 1.57 m/s sliding speed and at an applied load of 9.81 N. During the initial stage, for both sample-II and sample-III the wear rate is negative. This may be due to the fact that the surface of sample-II and sample-III and the rotating disc are in direct contact with the oxide layer. The oxide layer acts as a protective shield by preventing the metal-to-metal contact between the sliding surfaces and reduces the wear rate. The oxide layer is formed by the transfer of materials when sliding AMMC against steel counter face and thus increases the resistance to wear rate. However in case of sample-I, due to the absence of the protective oxide layer, sample-I is subjected to maximum wear. Comparing Figures 10(a), 11(a), and 12(a), it is clear that the worn surface of sample-II and sample-III is covered with near continuous oxide films compared to sample-I and coincides with the earlier findings on Al/SiCp composites [20]. It is pointed out in the literature that the COF decreases due to the formation of the oxide layer on the sample surface [21].

Figure 10(a) shows the SEM image of the worn surface of sample-I showing deep grooves formed due to the penetration of the hard asperities onto the soft pin surface. The presence of deep ploughing marks and delamination combined with severe plastic deformation confirms the evidence of adhesion wear. The formation of Mechanically Mixed Layer (MML) is found to be very low on the surface of sample-I and indicates that the rate of removal of oxide layer is severe compared to its formation. This may be due to the increase in the temperature caused due to friction, softening the matrix due to the lack of reasonable strength and hardness.

Among the three samples taken for the investigation, the hardness of sample-I is less compared with sample-II and sample-III. In case of sample-I, due to the lack of reasonable strength and hardness, the matrix gets easily softened at higher loads and sliding speed and prevents the formation of a stable protective oxide layer.  Figure 10(b) shows the XRD test report of sample-I. The XRD test report of sample-I reveals very small amount of Al_2_O_3_ and Fe_2_O_3_ compared with the other samples taken for the investigation. Thus due to lack of strong protective layer the wear rate of sample-I is very high and the wear rate would increase drastically if the sliding speed and sliding distance is further increased.

In this investigation, sample-II and sample-III have shown better wear resistance at higher load and sliding speed compared with sample-I. This may be due to the presence of uniformly distributed hard reinforcements like Al_2_O_3_, Si, ca, and Fe present in the WSD particles, increasing the hardness, thermal stability, and load bearing capacity of sample-II and sample-III. Thus the wear rate of sample-II and sample-III is less compared to sample-I. According to Shanthi et al. [22], the increase in the wear resistance of AZ1B/Al_2_O_3_-(1–3) wt% of Ca nanocomposite was attributed to the improved hardness and strength of the composite due to the presence of alumina and calcium (Ca) in the matrix coincides with our findings. Comparing Figures 10(a), 11(a), and 12(a), it is clear that at high loads and sliding distance, the worn tracks are deeper and in particular the surface of sample-I is characterized by deep grooves which indicates that the material removal is through microploughing, whereas in case of sample-II and sample-III, the material removal is by oxidation.

Figure 11(a) shows the SEM of the worn surface of sample-II. The worn surface of sample-II is observed to be uniform and covered with smaller size wear debris. The XRD test report of sample-II as shown in Figure 11(b) confirms strong protective oxide layer particles like Al_2_O_3_ and Fe_2_O_3_. It is pointed out in the literature that pulled-out hard reinforced particles from the matrix plays a major role in the material wear rate [19].

Figure 12(a) shows the SEM image of the worn surface of sample-III revealing shallow grooves running parallel to the sliding direction and ploughing marks and cracks indicate the main wear mechanism of sample-III as abrasive and adhesion. The presence of cracks in Figure 12(a) is probably due to pull-out of reinforcement particles due to reduced bonding strength between the reinforcement and the matrix at higher loads and sliding distance. Thus from the experimental results, it is clear that at high sliding velocity the oxide layer becomes thicker and brittle and can be broken down easily. The XRD test report of sample-III is shown in Figure 12(b). The peak shows the quantification of the mixtures present in sample-III. From Figure 12(a) it is clear that the MML gets fractured into agglomerated powders at higher load and sliding distances. The cracks can be seen propagating along the weaker region in MML and can be seen at the interference of MML and plastically deformed region.

**Figure 13:**
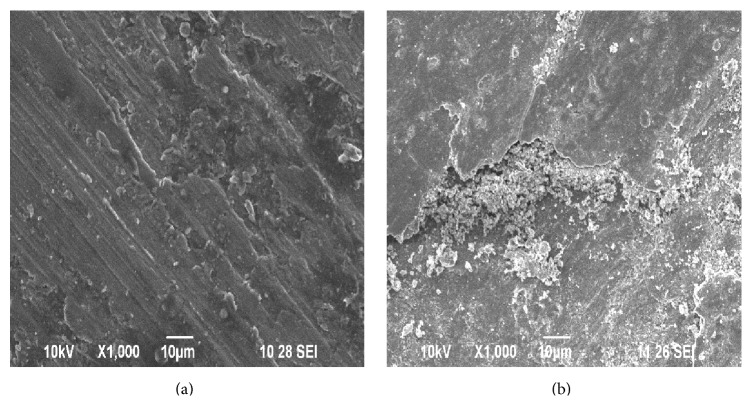
(a) SEM of sample-II at higher magnification before wear test. (b) SEM of wear track of sample-II at higher magnification after wear test (after running 2000 m at 29.43 N load and 4.71 m/s sliding speed).

Figures 13(a) and 13(b) show the SEM image of sample-II before and after the wear test at higher magnification. The presence of crack, broken oxide layer-flaky debris, and pores in the worn surface of sample-II shows signs of abrasive wear. It is pointed out in the literature that the pores may even impart beneficial effect on the wear resistance by trapping the wear debris inside the pores and reduce the possibility of particle agglomeration [23].

**Figure 14:**
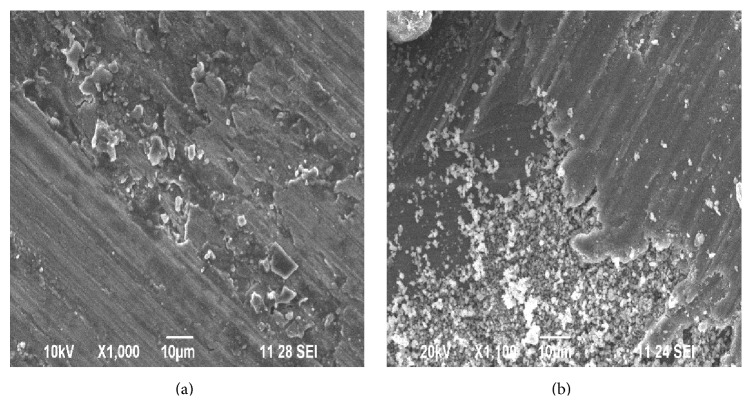
(a) SEM of sample-III at higher magnification before wear test. (b) SEM of wear track of sample-III at higher magnification after wear test (after running 3000 m at 29.43 N load and 4.71 m/s sliding speed).

Figures 14(a) and 14(b) show the SEM image of sample-III before and after the wear test at higher magnification. From the experimental results it can been seen that increasing the sliding distance increases the hardness of the work piece and in turn leads to fragmentation of the agglomerated powders to form smaller wear debris. According to [24], the removed oxide layer wear debris fills out the grooves on the pin surface and prevents the metal-to-metal contact. Thus the wear rate of sample-II and sample-III is comparatively less compared with that of sample-I even after running longer distance.

## 4. Conclusion

In this work low cost reinforced AMMC with improved wear resistance was being prepared by reinforcing WSD and thus the disposal/dumping of industrial waste and environmental pollution can be prevented. From the experimental results the following conclusions can be drawn from this work:

- Increasing the reinforcement content from 10 wt% to 20 wt% increases the hardness and wear resistance of the composites but reduces its ductility.
- The coefficient of friction was found to reduce with increase in the applied load.
- Oxide layer was found more evenly spread on both sample-II and sample-III, acts as a protective layer, prevents metal-to-metal contact, and reduces the wear rate, whereas the oxide layer was unstable and found to be very low on the surface of sample-I.
- Oxide layer formation was found to be very low on the surface of sample-I indicating that the rate of removal of oxide layer is severe compared to its formation due to low hardness and thus increases the wear rate of sample-I drastically.
- The oxide layer becomes thicker and brittle at higher load and sliding speed.

## Supplementary Material

The Supplementary material shows the recorded wear test report of the sample-I when the experiment was conducted at 9.81 N applied load, with 1.57 m/s sliding speed after running 1000 meter sliding distance. The following data was recorded: Time (Wear rate for every second), Wear rate, FF (Frictional Force) and Temperature. By using the friction force (F) value, the coefficient of friction was calculated by dividing the frictional force (F) by the normal load. Using the recorded data, a comparative graph can be created to compare the influence of each parameter on the wear rate.

## Figures and Tables

**Table 1:** Mechanical properties of the prepared composites.

Sl. number	Material	Hardness value (BHN)	Yield strength (MPa)	Ultimate tensile strength (MPa)	Elongation (%)
(1)	Sample-I	43	67	131	13
(2)	Sample-II	63	115	154	8.13
(3)	Sample-III	75	133	165	6.10
