# Supplementary material for: Wear Behavior of Aluminium Metal Matrix Composite Prepared from Industrial Waste
Source: ScientificWorldJournal. 2016 Feb 18;2016:6538345. doi: 10.1155/2016/6538345 (PMC4775816; doi:10.1155/2016/6538345)
Supplement: Supplementary file 1 — The Supplementary material shows the recorded wear test report of the sample-I when the experiment was conducted at 9.81 N applied load, with 1.57 m/s sliding speed after running 1000 meter sliding distance. The following data was recorded: Time (Wear rate for every second), Wear rate, FF (Frictional Force) and Temperature. By using the friction force (F) value, the coefficient of friction was calculated by dividing the frictional force (F) by the normal load. Using the recorded data, a comparative graph can be created to compare the influence of each parameter on the wear rate. [file 6538345.f1.doc]

TIME; WEAR; FF; TEMP

0.9090; -1.18; 0.88; 293.34

1.8190; -2.85; 2.17; 293.28

2.7280; -4.93; 3.10; 293.32

3.6370; -2.70; 3.37; 293.36

4.5470; -0.12; 3.46; 293.36

5.4560; 0.21; 3.52; 293.34

6.3660; 0.67; 3.56; 293.30

7.2750; 1.35; 3.11; 293.32

8.1850; 1.50; 2.27; 293.31

9.0950; 1.18; 1.95; 293.34

10.0040; 1.53; 2.26; 293.32

10.9140; 1.68; 2.68; 293.33

11.8220; 2.41; 2.91; 293.35

12.7320; 3.49; 3.09; 293.32

13.6430; 4.38; 3.20; 293.32

14.5520; 4.82; 3.30; 293.33

15.4620; 6.03; 3.43; 293.33

16.3710; 7.25; 3.48; 293.34

17.2810; 7.97; 3.44; 293.31

18.1900; 8.75; 3.47; 293.33

19.0990; 8.91; 3.53; 293.33

20.0090; 8.35; 3.50; 293.32

20.9180; 8.65; 3.35; 293.30

21.8280; 9.30; 3.23; 293.30

22.7370; 9.32; 3.20; 293.32

23.6460; 9.68; 3.26; 293.32

24.5560; 9.74; 3.34; 293.33

25.4650; 10.12; 3.28; 293.33

26.3760; 10.92; 3.15; 293.31

27.2840; 12.18; 3.06; 293.30

28.1940; 13.80; 3.09; 293.29

29.1030; 14.86; 3.04; 293.33

30.0130; 15.26; 2.84; 293.34

30.9230; 15.35; 2.75; 293.34

31.8330; 15.63; 2.79; 293.28

32.7420; 15.60; 2.85; 293.32

33.6520; 15.63; 2.90; 293.30

34.5610; 16.00; 2.91; 293.29

35.4710; 16.26; 2.89; 293.28

36.3800; 16.81; 2.85; 293.31

37.2900; 17.67; 2.75; 293.33

38.1990; 18.18; 2.68; 293.31

39.1090; 18.34; 2.69; 293.33

40.0180; 18.37; 2.73; 293.33

40.9270; 18.42; 2.80; 293.34

41.8370; 18.25; 2.87; 293.30

42.7460; 18.50; 2.90; 293.32

43.6550; 18.78; 2.87; 293.27

44.5650; 18.90; 2.83; 293.28

45.4740; 19.18; 2.77; 293.34

46.3840; 19.61; 2.72; 293.32

47.2930; 19.44; 2.67; 293.33

48.2030; 19.57; 2.67; 293.33

49.1140; 19.64; 2.68; 293.31

50.0230; 19.80; 2.61; 293.35

50.9330; 20.26; 2.56; 293.35

51.8420; 20.89; 2.55; 293.27

52.7510; 21.20; 2.56; 293.32

53.6610; 21.91; 2.58; 293.34

54.5700; 22.23; 2.60; 293.31

55.4800; 22.10; 2.65; 293.32

56.3890; 22.30; 2.70; 293.33

57.2990; 22.50; 2.73; 293.29

58.2080; 22.45; 2.76; 293.34

59.1180; 22.73; 2.84; 293.30

60.0270; 22.99; 2.89; 293.32

60.9360; 23.15; 2.96; 293.33

61.8450; 23.30; 2.99; 293.30

62.7550; 23.59; 2.96; 293.31

63.6640; 23.86; 2.94; 293.33

64.5740; 24.02; 2.97; 293.31

65.4830; 24.20; 3.01; 293.29

66.3940; 24.53; 2.97; 293.34

67.3030; 24.75; 2.85; 293.30

68.2140; 25.33; 2.82; 293.32

69.1220; 25.72; 2.80; 293.33

70.0320; 26.13; 2.80; 293.32

70.9410; 26.26; 2.87; 293.30

71.8510; 26.44; 2.90; 293.32

72.7600; 26.58; 2.85; 293.30

73.6700; 26.86; 2.83; 293.32

74.5800; 27.21; 2.80; 293.32

75.4890; 27.54; 2.81; 293.32

76.3980; 27.72; 2.87; 293.32

77.3080; 28.24; 2.96; 293.35

78.2170; 28.68; 3.03; 293.31

79.1270; 29.17; 3.02; 293.28

80.0360; 29.82; 2.98; 293.29

80.9450; 30.31; 2.94; 293.30

81.8550; 30.46; 2.89; 293.31

82.7640; 30.81; 2.86; 293.34

83.6750; 31.18; 2.85; 293.28

84.5840; 31.61; 2.91; 293.29

85.4930; 32.28; 2.97; 293.29

86.4030; 32.57; 2.98; 293.32

87.3120; 32.72; 2.91; 293.32

88.2220; 32.71; 2.85; 293.31

89.1310; 32.72; 2.86; 293.33

90.0410; 32.69; 2.81; 293.37

90.9510; 32.74; 2.73; 293.33

91.8600; 32.86; 2.69; 293.33

92.7700; 32.89; 2.70; 293.33

93.6790; 32.97; 2.71; 293.34

94.5880; 32.98; 2.67; 293.36

95.4980; 32.90; 2.65; 293.33

96.4070; 32.89; 2.73; 293.32

97.3170; 32.64; 2.84; 293.30

98.2260; 32.66; 2.89; 293.31

99.1360; 32.67; 2.90; 293.34

100.0450; 32.72; 2.89; 293.31

100.9540; 32.81; 2.93; 293.35

101.8650; 32.86; 2.93; 293.32

102.7740; 32.85; 2.89; 293.32

103.6830; 33.17; 2.79; 293.32

104.5930; 33.52; 2.66; 293.31

105.5020; 33.81; 2.60; 293.31

106.4120; 34.22; 2.60; 293.30

107.3210; 34.66; 2.62; 293.31

108.2310; 34.98; 2.64; 293.33

109.1400; 35.24; 2.67; 293.30

110.0500; 35.44; 2.75; 293.32

110.9590; 35.67; 2.81; 293.31

111.8690; 36.05; 2.78; 293.33

112.7780; 36.65; 2.73; 293.32

113.6870; 37.13; 2.76; 293.32

114.5970; 37.35; 2.77; 293.32

115.5070; 37.54; 2.79; 293.30

116.4160; 37.47; 2.78; 293.32

117.3260; 37.25; 2.79; 293.27

118.2350; 37.59; 2.82; 293.33

119.1460; 38.05; 2.82; 293.31

120.0550; 38.33; 2.82; 293.36

120.9640; 38.60; 2.87; 293.31

121.8740; 38.67; 2.92; 293.31

122.7830; 38.80; 2.98; 293.33

123.6930; 38.81; 2.98; 293.31

124.6020; 38.81; 3.01; 293.30

125.5120; 39.09; 3.03; 293.31

126.4210; 39.32; 3.01; 293.31

127.3300; 39.46; 3.01; 293.30

128.2400; 39.84; 2.99; 293.36

129.1490; 40.11; 2.94; 293.34

130.0590; 40.32; 2.89; 293.32

130.9690; 40.42; 2.86; 293.32

131.8780; 40.43; 2.83; 293.38

132.7880; 40.68; 2.84; 293.33

133.6970; 40.58; 2.85; 293.33

134.6070; 40.60; 2.87; 293.30

135.5160; 40.82; 2.90; 293.32

136.4260; 40.93; 2.92; 293.27

137.3360; 41.09; 2.91; 293.32

138.2450; 41.38; 2.93; 293.31

139.1550; 41.65; 2.96; 293.29

140.0640; 41.93; 2.93; 293.31

140.9740; 42.05; 2.88; 293.31

141.8830; 42.16; 2.88; 293.29

142.7920; 42.32; 2.89; 293.32

143.7020; 42.44; 2.90; 293.34

144.6110; 42.64; 2.90; 293.28

145.5200; 42.70; 2.84; 293.30

146.4300; 42.55; 2.76; 293.33

147.3390; 42.45; 2.73; 293.33

148.2500; 42.45; 2.74; 293.34

149.1580; 42.29; 2.71; 293.31

150.0690; 42.71; 2.78; 293.32

150.9770; 43.33; 2.98; 293.34

151.8870; 43.68; 3.13; 293.36

152.7970; 43.96; 3.08; 293.32

153.7070; 44.03; 2.93; 293.32

154.6170; 43.95; 2.84; 293.33

155.5260; 44.01; 2.88; 293.33

156.4350; 44.11; 2.94; 293.32

157.3450; 44.54; 2.93; 293.30

158.2540; 44.76; 2.90; 293.33

159.1640; 45.11; 2.85; 293.29

160.0730; 45.27; 2.81; 293.33

160.9830; 45.61; 2.83; 293.34

161.8920; 45.73; 2.87; 293.31

162.8010; 45.96; 2.89; 293.33

163.7110; 46.08; 2.87; 293.33

164.6200; 46.05; 2.82; 293.33

165.5300; 46.01; 2.78; 293.33

166.4390; 46.12; 2.76; 293.33

167.3480; 46.37; 2.77; 293.32

168.2580; 46.73; 2.83; 293.27

169.1670; 46.89; 2.89; 293.31

170.0770; 47.00; 2.94; 293.32

170.9870; 47.01; 2.94; 293.33

171.8970; 47.06; 2.87; 293.32

172.8070; 47.09; 2.86; 293.31

173.7160; 47.19; 2.91; 293.31

174.6260; 47.45; 2.87; 293.35

175.5350; 47.38; 2.84; 293.33

176.4450; 47.15; 2.88; 293.32

177.3540; 47.10; 2.95; 293.32

178.2630; 47.07; 2.98; 293.31

179.1730; 47.21; 3.02; 293.29

180.0820; 47.86; 3.08; 293.31

180.9920; 48.25; 3.12; 293.32

181.9010; 48.73; 3.12; 293.31

182.8110; 49.18; 3.10; 293.29

183.7200; 49.21; 3.07; 293.31

184.6290; 49.26; 3.03; 293.30

185.5380; 49.21; 2.98; 293.32

186.4480; 49.18; 2.95; 293.33

187.3580; 49.07; 2.94; 293.33

188.2680; 49.33; 2.94; 293.35

189.1770; 49.70; 2.95; 293.32

190.0880; 50.18; 2.92; 293.33

190.9960; 50.75; 2.82; 293.33

191.9070; 51.20; 2.75; 293.33

192.8150; 51.73; 2.75; 293.34

193.7250; 52.06; 2.82; 293.32

194.6350; 52.30; 2.88; 293.32

195.5440; 52.43; 2.90; 293.38

196.4540; 51.98; 2.84; 293.28

197.3630; 51.45; 2.75; 293.32

198.2730; 50.89; 2.70; 293.34

199.1820; 50.25; 2.68; 293.35

200.0910; 49.85; 2.67; 293.33

201.0010; 49.66; 2.64; 293.33

201.9100; 49.44; 2.65; 293.34

202.8190; 49.33; 2.69; 293.32

203.7290; 49.38; 2.71; 293.32

204.6380; 49.58; 2.72; 293.34

205.5480; 49.83; 2.75; 293.35

206.4580; 49.85; 2.73; 293.33

207.3680; 49.82; 2.69; 293.34

208.2770; 49.62; 2.67; 293.32

209.1870; 49.45; 2.68; 293.30

210.0960; 49.56; 2.71; 293.33

211.0050; 50.04; 2.75; 293.33

211.9160; 50.49; 2.78; 293.31

212.8240; 50.93; 2.81; 293.32

213.7340; 51.51; 2.84; 293.36

214.6440; 52.00; 2.86; 293.27

215.5530; 52.11; 2.89; 293.33

216.4630; 52.19; 2.89; 293.34

217.3720; 52.41; 2.87; 293.35

218.2810; 52.36; 2.84; 293.35

219.1910; 52.26; 2.83; 293.34

220.1000; 52.44; 2.85; 293.32

221.0100; 52.62; 2.90; 293.31

221.9190; 52.81; 2.91; 293.34

222.8290; 52.96; 2.90; 293.35

223.7390; 53.22; 2.89; 293.35

224.6480; 53.63; 2.93; 293.31

225.5580; 53.91; 2.97; 293.34

226.4670; 54.38; 3.01; 293.32

227.3770; 54.82; 3.04; 293.30

228.2860; 55.15; 3.04; 293.34

229.1950; 55.39; 3.05; 293.32

230.1060; 55.46; 3.02; 293.32

231.0140; 55.53; 2.99; 293.33

231.9250; 55.55; 2.95; 293.35

232.8330; 55.40; 2.95; 293.35

233.7430; 55.15; 2.97; 293.32

234.6530; 55.19; 2.95; 293.31

235.5620; 54.99; 2.89; 293.34

236.4720; 54.88; 2.88; 293.35

237.3810; 55.07; 2.88; 293.35

238.2910; 55.13; 2.86; 293.33

239.2000; 55.02; 2.85; 293.35

240.1100; 55.19; 2.83; 293.30

241.0200; 55.43; 2.79; 293.35

241.9290; 55.66; 2.75; 293.36

242.8390; 55.80; 2.73; 293.32

243.7480; 56.16; 2.72; 293.34

244.6570; 56.48; 2.73; 293.34

245.5670; 56.71; 2.72; 293.34

246.4760; 57.10; 2.69; 293.34

247.3860; 57.15; 2.69; 293.34

248.2950; 57.15; 2.70; 293.32

249.2040; 57.37; 2.74; 293.31

250.1140; 57.06; 2.90; 293.31

251.0230; 56.56; 3.04; 293.35

251.9330; 56.42; 3.32; 293.32

252.8420; 55.96; 3.94; 293.34

253.7530; 55.38; 4.21; 293.35

254.6620; 56.47; 3.76; 293.33

255.5720; 57.68; 3.20; 293.31

256.4810; 58.41; 2.87; 293.35

257.3900; 59.31; 2.70; 293.35

258.3000; 59.42; 2.60; 293.34

259.2100; 58.77; 2.51; 293.36

260.1200; 58.05; 2.48; 293.36

261.0290; 58.15; 2.46; 293.32

261.9380; 58.10; 2.47; 293.32

262.8480; 58.35; 2.49; 293.32

263.7570; 58.62; 2.52; 293.33

264.6670; 59.02; 2.59; 293.34

265.5760; 58.93; 2.68; 293.34

266.4850; 58.96; 2.68; 293.34

267.3950; 58.93; 2.63; 293.35

268.3040; 58.56; 2.65; 293.34

269.2130; 58.44; 2.66; 293.29

270.1230; 58.52; 2.63; 293.39

271.0330; 58.63; 2.66; 293.37

271.9430; 59.07; 2.73; 293.34

272.8510; 59.39; 2.77; 293.35

273.7620; 59.73; 2.75; 293.32

274.6710; 59.94; 2.76; 293.35

275.5810; 60.14; 2.82; 293.37

276.4910; 60.21; 2.90; 293.35

277.4000; 60.56; 2.91; 293.34

278.3100; 60.57; 2.90; 293.34

279.2200; 60.78; 2.93; 293.35

280.1290; 61.08; 2.96; 293.33

281.0380; 61.31; 2.95; 293.32

281.9470; 61.37; 2.92; 293.34

282.8570; 61.44; 2.91; 293.36

283.7660; 61.29; 2.94; 293.33

284.6760; 61.05; 2.93; 293.32

285.5850; 61.15; 2.88; 293.34

286.4950; 61.34; 2.87; 293.32

287.4040; 61.66; 2.81; 293.34

288.3130; 62.21; 2.79; 293.35

289.2230; 62.61; 2.75; 293.35

290.1320; 62.71; 2.68; 293.39

291.0420; 62.82; 2.65; 293.32

291.9510; 62.93; 2.66; 293.31

292.8600; 63.17; 2.67; 293.39

293.7720; 63.44; 2.67; 293.32

294.6810; 63.59; 2.69; 293.33

295.5910; 63.56; 2.72; 293.36

296.5000; 63.38; 2.72; 293.34

297.4100; 63.16; 2.68; 293.36

298.3190; 62.63; 2.68; 293.35
